# High-performance Acetone Soluble Tape Transfer Printing Method for Heterogeneous Integration

**DOI:** 10.1038/s41598-019-52235-0

**Published:** 2019-10-31

**Authors:** Jiaqi Zhang, Yichang Wu, Zhe Li, Yachao Zhang, Yue Peng, Dazheng Chen, Weidong Zhu, Shengrui Xu, Chunfu Zhang, Yue Hao

**Affiliations:** State Key Discipline Laboratory of Wide Band Gap Semiconductor Technology, School of Microelectronics, Xidian University, 2 South Taibai Road, Xi’an, 710071 China

**Keywords:** Electrical and electronic engineering, Electronic devices

## Abstract

A high-performance transfer printing method using a new soluble tape which can be dissolved in acetone is proposed to be used in heterogeneous integration. Si inks array was transferred from SOI wafers onto various substrates without adhesion promoter by this new method which we refer to as the acetone soluble tape (AST) method to compare with other transfer printing methods by using thermal release tape (TRT), water soluble tape (WST) and polydimethylsiloxane (PDMS). By using the AST method, the transfer printing process does not involve interface contention between stamp/inks and inks/receiver substrate so that it maximizes the transfer printing efficiency. Experimental results present the AST method has good performances, and various alien substrates, even curvilinear surfaces, can be selected as receiver substrates by the AST method. To examine the quality of the transferred Si inks, the Si TFTs were fabricated by using the Si membrane transferred by the AST method on sapphire substrate and the devices show the good performance. All the results confirm that the AST method is an effective method in heterogeneous integration.

## Introduction

Recently, there is growing interest in heterogeneous integration. Integrating different materials on one single chip to fabricate different devices (e.g. GaN and Ga_2_O_3_ can be used to fabricate power devices, GaAs can be used to fabricate high-frequency devices, and Si can be used to fabricate digital control circuitry) is a key means to increase the integration scale and functional diversity of chip^1,2^. Transfer printing is a widely adopted method for heterogeneous integration, typically, by using PDMS^3–7^, thermal release tape (TRT)^8^, water soluble tape (WST)^9^ and laser-driven non-contact transfer printing^10–12^. PDMS is a widely used transfer printing method which is accepted by people. TRT is the thermal release tape which will lose adhesion at a certain temperature. And inks on it can be released onto receiver substrates at releasing temperature. WST is the water soluble tape whose adhesive can dissolve in water. And inks on it can be released onto receiver substrates in water. Laser-driven non-contact transfer printing is the only transfer printing technique that can be manipulated in a non-contact printing mode reported so far.

Due to high temperature process existing in impurity activation and alloying annealing which are essential process steps in microelectronics industry, adhesion promoter which can’t bear the high temperature shouldn’t be coated on receiver substrates. Hence, transfer printing using PDMS by kinetic control of adhesion without adhesion promoter is developed and applied^13–15^. But there are two factors in this approach limiting its use: (1) the adhesion strength which depends on peeling velocity is difficult to control during “pick-up” and “printing”; (2) the adhesion switching ratio is too low (i.e., ~3) to complete an effective transfer printing^16^. Then some efforts were reported including modifying the surface of PDMS to increase the adhesion switching ratio^16,17^ and changing the operation method of transfer printing^18,19^. These improvements were proved to be effective to increase the transfer printing efficiency. However, the method which is modifying the surface of PDMS is high-cost, complicated and device-dependent. And the method which is changing the operation of transfer printing is equipment-dependent and increases the operation difficulty of transfer printing.

In this work, we explored a simple, low-cost, high-performance transfer printing method, namely, AST transfer printing method which can transfer printing inks without adhesion promoter. The adhesive and liner of AST both can be dissolved in acetone so that there isn’t interface contention between stamp/inks and inks/receiver substrate during “printing” process, so it can maximize the transfer printing efficiency. And the liner of AST is inelastic so that it can maintain the exact arrangement of inks to realize high fidelity. By this method, inks can be transferred on variety of substrates. Furthermore, inks can be transferred and printed on curvilinear surfaces by transfer printing using AST. It can be used in heterogeneous integration. And this new method may integrate various functional materials onto one substrate to fabricate a high-performance chip with high-integration density and versatile electronic systems. What’s more, it is an effective way to continue Moore’s law.

## Results and Discussions

### The transfer printing process of AST

**Figure 1:**
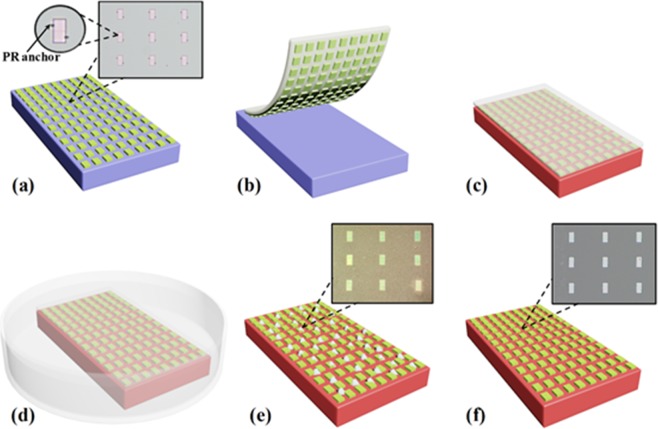
Schematic illustration of transfer printing process of AST. (**a**) Si inks array undercut etched on bottom silicon substrate and fixed by PR anchors. (**b**) Si inks array picked up by AST. (**c**) Coupling Si inks array with receiver substrate. (**d**) Immersing the coupling system in acetone. (**e**) Si inks array transferred on receiver substrate. (**f**) Receiver substrate after O_2_ plasma process.

SOI wafer was cleaned by acetone, alcohol and DI water. Si inks array was formed by lithography and RIE on SOI wafer. The wafer was immersed in buffer oxide etchant (BOE, 1:6) to etch the part of exposed buried oxide layer (BOX) for 10 minutes. Then using lithography fabricated PR anchors on the wafer. Immersing the wafer in concentrated hydrofluoric acid (HF, 49%) entirely removed the rest of BOX which is under Si inks array for 2 hours so that the Si inks array dropped on the bottom silicon substrate by Van der Waals force^20^. Due to PR anchors, Si inks array which was complete undercut etched wasn’t dislocated or scattered. Figure 1a–f show the main transfer printing process of AST. Figure 1a shows that Si inks array which was complete undercut etched on the bottom silicon substrate and was fixed by PR anchors. Then Si inks array was picked up by AST from bottom silicon substrate, as illustrated in Fig. 1b. Owing to the strong adhesive strength of AST, it can overcome Van der Waals force between Si inks array and bottom silicon substrate and crack the PR anchors from the edges of Si inks array so that Si inks array can be acquired by AST. Any wafers whose surface is flat could be selected as receiver substrates. For example, GaN/Sapphire substrate was selected as a receiver substrate. Coupling AST which acquired Si inks array with receiver substrate by appropriate laminating formed strong adhesive strength between them, as shown in Fig. 1c. The coupling system was fully immersed in acetone, as shown in Fig. 1d. AST was dissolved in acetone about 5–10 minutes. Then the receiver substrate onto which Si inks array was printed was cleaned by DI water, as shown in Fig. 1e. It is obvious from the optical image that there are lots of tape residuals on receiver substrate and Si inks array. Because tape residuals directly affect the performance of electronic devices, they must be fully removed. Hence, the receiver substrate on which Si inks array was printed was treated with O_2_ plasma (300 W, 300 sccm) for 10 minutes so that the tape residuals were fully removed, as shown in Fig. 1f. Optical image shows that tape residuals were removed.

### Extraction and analysis of key parameters of transfer printing methods

**Figure 2:**
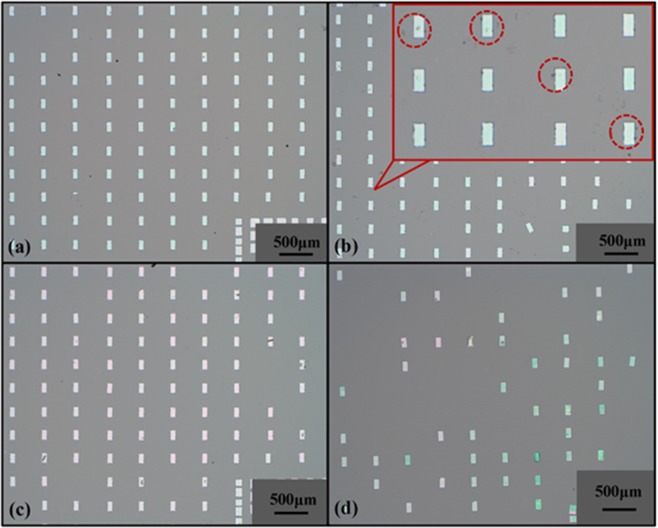
Optical images of Si inks array transferred onto GaN/Sapphire substrates by four methods. (**a**–**d**) Optical image of Si inks array on GaN/Sapphire substrates by AST, TRT, WST and PDMS, respectively.

To confirm the transfer printing performance of AST, a comparative experiment using different transfer printing methods which are AST, TRT, WST and PDMS to transfer Si inks array onto GaN/Sapphire substrates without adhesion promoter. Figure 2a–d show the optical images of Si inks array on GaN/Sapphire substrates by these four methods, respectively. Figures 2a,c shows a better transfer printing quality, by AST and WST. There are some tape residuals on inks and receiver substrate by TRT, which can degrade the devices properties, as shown in Fig. 2b. Figure 2d shows a low transfer printing efficiency by PDMS.

**Figure 3:**
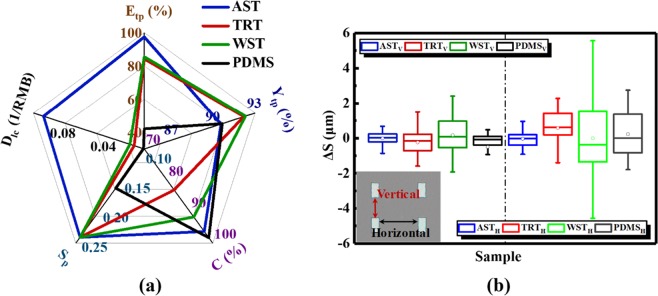
Contrastive plots of the critical parameters of four transfer printing methods. (**a**) Radar plot shows the transfer printing efficiency (E_tp_), yield (Y_tp_), cleanliness (C), process simplicity (S_p_) and low-cost degree (D_lc_) of four transfer printing methods. (**b**) Box plot shows the location shift (ΔS) of Si inks array transferred onto GaN/Sapphire substrate through four transfer printing methods.

Figure 3a shows the critical properties of four transfer printing methods. There are 152 ink elements on each donor substrate. Transfer printing efficiency (E_tp_) was extracted basing on the equation ${E}_{tp}=({n}_{1}/{n}_{0})100 \%$. “n_1_” is the number of Si inks which are transferred onto receiver substrate. “n_0_” represents the number of Si inks on donor substrate. AST has the highest E_tp_ up to 97.37% due to the printing process which doesn’t involve interface contention. E_tp_ of TRT and WST are not much different from each other, 84.21% and 85.53%, respectively. As was expected, E_tp_ of PDMS is the lowest, which just is 42.11%, even though Si inks were transferred using PDMS by rapidly picking up (≥10 cm/s) and slowly printing (≤1 mm/s)^17^. Transfer printing yield (Y_tp_) was extracted basing on the equation ${Y}_{tp}=({n}_{2}/{n}_{1})100 \%$. “n_2_” is the number of Si inks which are intactly transferred onto receiver substrate without cracks or wrinkles. Y_tp_ of AST, TRT, WST and PDMS are all above 90%. Y_tp_ of AST and PDMS are on the similar level, 90.54% and 90.63%. TRT and WST have higher transfer printing yields, 92.19% and 92.31%, respectively. If the thickness of liner of tape is small, the tape won’t have a good buffer function for inks during transfer printing. The liner of AST is a little thinner than TRT and WST. And AST protects inks less than TRT and WST. Si inks were susceptible to damage during transfer printing. Therefore, the transfer printing yield of AST is a little lower than that of TRT and WST. Any residuals on inks during transfer printing process can degrade or even invalidate devices. Therefore, Cleanliness is also an important parameter to evaluate the quality of a transfer printing method. In this study, cleanliness (C) is derived from the equation ${\rm{C}}=({n}_{3}/{n}_{2})100 \%$. “n_3_” is the number of Si inks which are clean without tape residuals in “n_2_”. Cleanliness of PDMS is the highest up to 98.27%. PDMS is elastomeric and its fabrication process is extremely clean, so there are almost no residuals left on inks or receiver substrate after transfer printing. Cleanliness of AST is next to that of PDMS, up to 96.27%. Although there was still most of adhesive (tape residuals) which isn’t dissolved after being immersed in acetone left on inks and receiver substrate, it can be almost removed completely by O_2_ plasma process so that AST method has a high cleanliness. WST’s cleanliness is up to 91.67%. TRT’s cleanliness is the lowest, just 83.05%, due to certain residuals which can’t be removed by O_2_ plasma or Piranha solution. Process simplicity (S_p_) is derived from the reciprocal of number of transfer printing process steps. The transfer printing processes of AST, TRT and WST all involve four steps, “picking up”, “coupling”, “releasing” and “removing residuals”. And the only difference between the three methods is the way of “releasing”. “Releasing” of AST is in acetone, “releasing” of TRT is on hot plate and “releasing” of WST is in DI water. However, step number of PDMS is six, including “cleaning mold”, “preparation of PDMS”, “curing PDMS”, “cutting PDMS”, “picking up” and “printing”. In addition, “picking up” and “printing” are two difficult steps due to the adhesion strength which is controlled by peeling velocity. Low-cost degree (D_lc_) is derived from the reciprocal of unit price of the transfer printing stamps. TRT and WST have similar prices about 100 RMB. PDMS needs 1200 RMB. However, AST just costs about 10 RMB. Therefore, if AST was used in heterogeneous integration field, it will reduce the cost greatly.

If inks are dislocated seriously after transfer printing, it will lead to alignment errors which can induce that the characteristics of the devices are asymmetric or devices failure. Hence, fidelity is another important parameter for evaluating transfer printing methods. In this work, fidelity is represented by location shift (ΔS). ΔS is divided into two categories, vertical location shift (ΔS_V_) and horizontal location shift (ΔS_H_), as shown in the optical image of Fig. 3b. Figure 3b shows box plot of ΔS_V_ and ΔS_H_ of four transfer printing methods. There is an average of 60 data points in each group of this eight groups. As shown in the figure, whichever method is used, there is still existing location shift. The vast majority of ΔS_V_ and ΔS_H_ of all methods are within ±2 μm. ΔS_V_ and ΔS_H_ of AST are both minimum (|ΔS_V_|, |ΔS_H_|<0.5 μm) and distributed uniform around zero. All the |ΔS_H_| is larger than |ΔS_V_| (|ΔS_H_|>|ΔS_V_|) in each method, because stamps will suffer from slight tensile strain inducing the increase of |ΔS_H_| when stamps pick inks up in horizontal direction. And positive values of ΔS_H_ is more than negative values of ΔS_H_ in most of methods due to the tensile strain which can stretch the stamps in horizontal direction. But positive and negative values of AST’s ΔS_H_ are distributed uniform, possibly due to a better stretching resistance of AST.

### Study on the universality of AST

**Figure 4:**
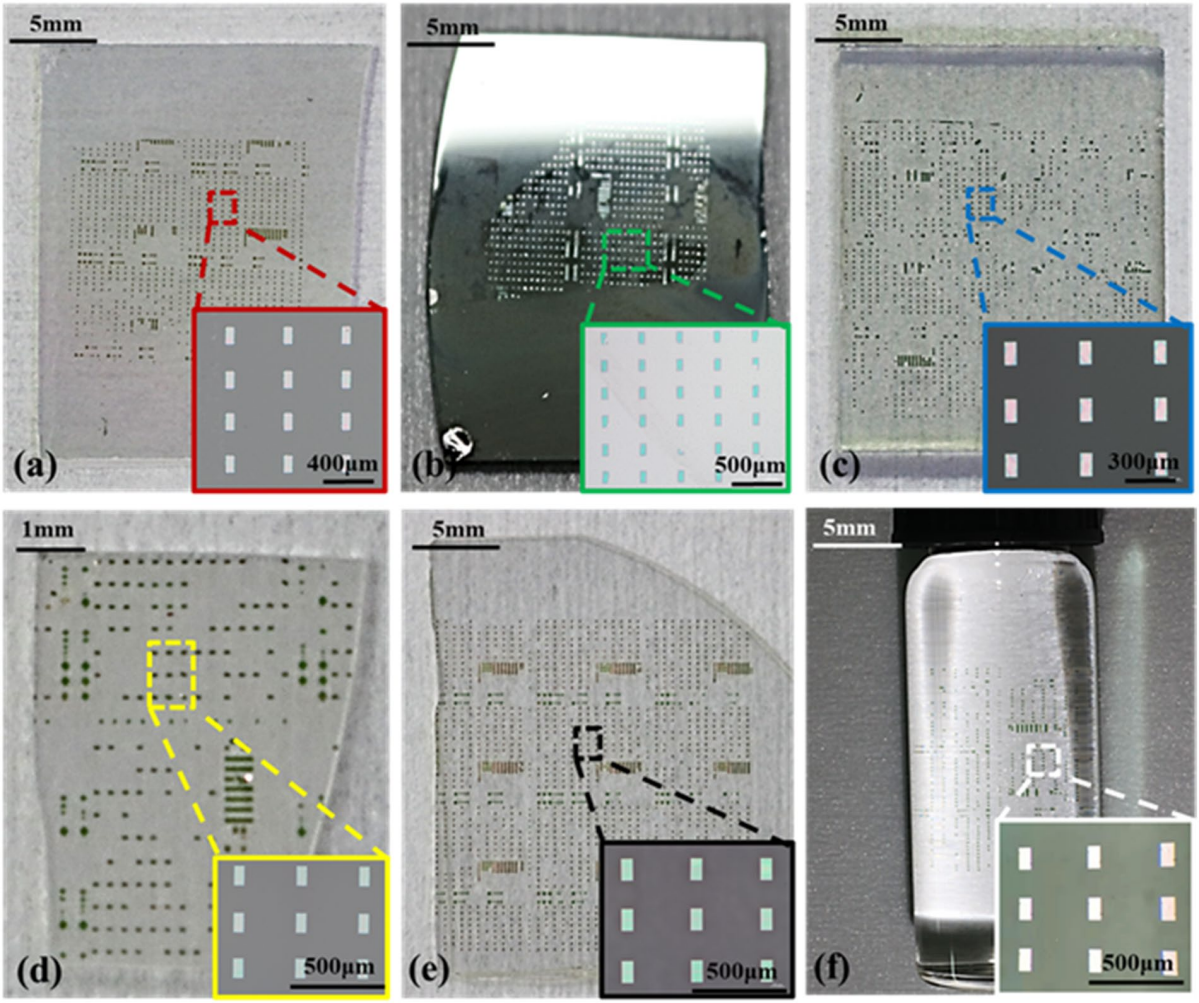
Si inks array transferred onto various alien substrates by AST. (**a**) AlGaN/GaN/Sapphire substrate. (**b**) Ge substrate. (**c**,**d**) FTO substrate and Ga_2_O_3_ substrate which weren’t processed by O_2_ plasma, respectively. (**e**) Sapphire substrate (Al_2_O_3_) which was processed by O_2_ plasma. (**f**) Glass cylinder.

Figure 4a–f show that Si inks array was transferred onto various alien substrates by AST. Figure 4a shows that Si inks array was transferred onto AlGaN/GaN/Sapphire substrate (E_tp_ = 95.63%). Figure 4b shows Si inks array was printed onto Ge substrate (E_tp_ = 93.42%). The transfer printing method of AST doesn’t work well on oxide substrates. It hasn’t been known what reason induces these results. As shown in Fig. 4c,d, Si inks array was printed onto FTO substrate and Ga_2_O_3_ substrate. And E_tp_ of AST on them are both low, 57.89% and 62.73%, respectively. But improvement can be realized by using O_2_ plasma process which can produce lots of suspension bonds on oxide substrates. These suspension bonds can make Si inks and oxide substrates bond more robustly. As shown in Fig. 4e, Si inks array was transferred onto sapphire substrate (Al_2_O_3_) which was processed by O_2_ plasma. Its E_tp_ is up to 90.82%. Moreover, Si inks array also can be transferred onto curvilinear surfaces. As shown in Fig. 4f, Si inks array was printed onto a glass cylinder whose radius is 0.5 cm. The illustration is a photograph taken under a metallographic microscope.

### Silicon devices were prepared by AST method

**Figure 5:**
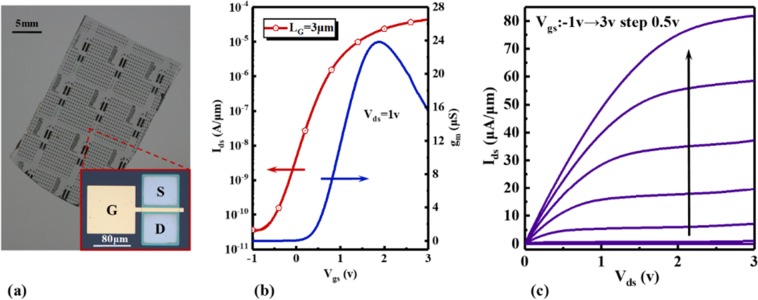
Devices and its electrical characteristics. (**a**) Si TFTs fabricated on sapphire substrate by AST. (**b**) The transfer characteristics of Si TFTs. (c) I-V characteristics of Si TFTs.

Finally, to confirm that devices based on AST method can be used in heterogeneous integration, we fabricate Si TFTs on sapphire substrate by AST. Figure 5a shows the picture of Si TFTs on sapphire substrate and optical images of one device. Figure 5b shows the transfer characteristics of TFTs. The gate length is 3 μm, and I_on/off_ is up to 10^6^. The peak transconductance is 24 μS and the threshold voltage is 1.03 V. Figure 5c presents I-V characteristics of one device. The low resistance (0.81 Ω·mm) of the ohmic contacts was extracted in these devices.

## Conclusions

A new transfer printing method which can be used in heterogeneous integration field was discovered by using AST. Because AST can be dissolved in acetone, the transfer printing process doesn’t involve interface contention between stamp/inks and inks/receiver substrate so that it maximizes the transfer printing efficiency. AST has satisfactory performances in E_tp_, Y_tp_, C, S_p_, D_lc_ and ΔS. And it also can transfer Si inks onto various alien substrates, even curvilinear objects. Although it doesn’t work well on oxide substrates, it can be improved by surface treatment on these substrates. Si TFTs can be fabricated on sapphire substrate by AST method. All the results indicate that AST is an effective method in heterogeneous integration field.

## Methods

### Preparation of PDMS

A flat silicon wafer was clean by acetone, alcohol, DI water and piranha solution. 10:1 PDMS stamp was cast on this silicon wafer in petri dishes. PDMS stamp was cured at 65 °C for 4 hours. The thickness of PDMS stamp is about 7 mm. Finally, PDMS stamp was cut into 2 cm × 4 cm dices.

### Fabrication of Si TFTs on sapphire

Silicon-on-insulator wafer (SOI) (Soitec by Smartcut with 200 nm top Si which is doped boron whose level is 8 × 10^14^ cm^−3^ and 200 nm buried oxide) was selected as a donor substrate. The phosphorus ion implantation energy and dose are 30 keV and 5 × 10^15^ cm^−2^, respectively. The dopants were activated by RTP at 900 °C for 30 s in N_2_. Si inks array was transferred onto sapphire substrate by AST method. Si inks and sapphire substrate will bond robustly by RTP at 500 °C for 3 min. 10 nm Al_2_O_3_ film was grown by ALD as the gate dielectric and 20 nm Ti followed by 120 nm Au were used as the gate electrode. Vias were opened by BOE in the S/D regions. And 30 nm Ni was deposited by e-beam evaporation followed by RTP at 200 °C for 30 s in N_2_.
